# Therapeutic efficacy of alternative primaquine regimens to standard treatment in preventing relapses by Plasmodium vivax: A systematic review and meta-analysis

**Published:** 2015-12-30

**Authors:** Lina Marcela Zuluaga-Idarraga, María-Eulalia Tamayo Perez, Daniel Camilo Aguirre-Acevedo

**Affiliations:** 1 Grupo Malaria, Facultad de Medicina. Universidad de Antioquia. Medellín, Colombia.; 2 Grupo Epidemiología y Bioestadística, Facultad de Medicina. Universidad CES. Medellín, Colombia Medellín,; 3 Grupo Académico de Epidemiología Clínica, Facultad de Medicina. Universidad de Antioquia. Medellín, Colombia.; 4 Departamento de Pediatría, Universidad de Antioquia, Fundación Hospital Universitario San Vicente, Medellín, Colombia.

**Keywords:** Malaria, Plasmodium vivax, pharmacovigilance, primaquine, recurrence

## Abstract

**Objective:**

To compare efficacy and safety of primaquine regimens currently used to prevent relapses by *P. vivax.*

**Methods:**

A systematic review was carried out to identify clinical trials evaluating efficacy and safety to prevent malaria recurrences by *P. vivax* of primaquine regimen 0.5 mg/kg/ day for 7 or 14 days compared to standard regimen of 0.25 mg/kg/day for 14 days. Efficacy of primaquine according to cumulative incidence of recurrences after 28 days was determined. The overall relative risk with fixed-effects meta-analysis was estimated.

**Results:**

For the regimen 0.5 mg/kg/day/7 days were identified 7 studies, which showed an incidence of recurrence between 0% and 20% with follow-up 60-210 days; only 4 studies comparing with the standard regimen 0.25 mg/kg/day/14 days and no difference in recurrences between both regimens (RR= 0.977, 95% CI= 0.670 to 1.423) were found. 3 clinical trials using regimen 0.5 mg/kg/day/14 days with an incidence of recurrences between 1.8% and 18.0% during 330-365 days were identified; only one study comparing with the standard regimen (RR= 0.846, 95% CI= 0.484 to 1.477). High risk of bias and differences in handling of included studies were found.

**Conclusion:**

Available evidence is insufficient to determine whether currently PQ regimens used as alternative rather than standard treatment have better efficacy and safety in preventing relapse of *P. vivax*. Clinical trials are required to guide changes in treatment regimen of malaria vivax.

## Abstract (es)

**Objetivo:**

Comparar la eficacia y seguridad de los esquemas de primaquina actualmente usados para prevenir las recaídas de malaria por *P. vivax*.

**Métodos:**

A través de una revisión sistemática se identificaron ensayos clínicos que evaluaran la eficacia y seguridad para prevenir recurrencias por *P. vivax *del régimen de primaquina 0.5 mg/Kg/día por 7 o 14 días comparado al régimen estándar de 0.25 mg/Kg/día por 14 días. Se determinó la eficacia de primaquina con la incidencia acumulada de recurrencias posterior a 28 días. Se estimó el riesgo relativo global con un meta-análisis de efectos fijos.

**Resultados:**

Se identificaron 7 ensayos clínicos para el régimen 0.5 mg/Kg/día/7 días que mostraron una incidencia de recurrencias entre 0% y 20% con un seguimiento de 60 a 210 días; solo 4 estudios compararon con el régimen estándar y no se encontraron diferencias en las recurrencias entre ambos esquemas (RR= 0.977; IC 95%= 0.670-1.423). Se identificaron tres ensayos clínicos que usaron el esquema 0.5 mg/Kg/día/14 días con una incidencia de recurrencias entre 1.8% y 18.0% para 330 a 365 días; solo un estudio comparó con el régimen estándar (RR= 0.846; IC 95%= 0.484-1.477). Se encontró alto riesgo de sesgo y diferencias en la conducción de los estudios incluidos.

**Conclusión:**

No hay suficiente evidencia para determinar si los regímenes de primaquina usados como alternativas al tratamiento estándar tienen mejor eficacia para prevenir las recaídas de *P. vivax*. Se requieren ensayos clínicos para orientar los cambios en el esquema de tratamiento de este tipo de malaria.

## Introduction 

Malaria caused by *Plasmodium vivax* is a major public health problem; about 20 million cases of malaria worldwide occur by this species, mainly in Asia and The Americas 1, 2. This species is characterized by the formation of a hepatic stage known as hipnozoite that lies dormant in the liver and can reactivate causing a relapse after treatment and cure of primary episode 3. Different relapse patterns are acknowledged for *P. vivax*, but two are prevailing 1) The pattern of tropical regions having a first relapse early and later relapses in short time intervals, usually every month; this pattern prevails in South America, Southeast Asia and Oceania 2. The predominant pattern in mild regions in Europe and Asia has a first late relapse usually between 8 and 10 months later and later short-term episodes 3-5. Although it is unclear what causes the reactivation of hypnozoites, this phenomenon is one of the main obstacles to the elimination of malaria, since they maintain active transmission of the disease 2,6.

For the treatment of *P. vivax*, the combination of two antimalarials is used: A blood schizontocidal that eliminates blood forms and tissue schizontocide that eliminates liver forms 7. There are several antimalarials targeting the blood forms of *P. vivax*, such as chloroquine, amodiaquine, artesunate, artemether, lumefantrine, dihydroartemisinin, piperaquine, chloroquine remains the most widely used as first-line treatment for this species 2,7. Primaquine (PQ) is the only antimalarial available to treat tissue forms and prevent relapses of *P. vivax* 2. 

In order to evaluate the therapeutic efficacy of treatment regimens for *P. vivax*, these must be administered and supervised so patients must be monitored during the first post-treatment month in order to ensure the cure of the primary attack and then monitor relapses 8. Usually these studies are conducted in endemic regions where relapses and reinfections (a new reinfection from the mosquito bite) can occur simultaneously and are indistinguishable by conventional diagnostic methods; therefore, the assessment of the therapeutic efficacy of the PQ has been measured in terms of recurrences including both relapses and reinfections 9,10. 

The World Health Organization (WHO) recommends the use of PQ under the regimen 0.25 mg/kg/day for 14 days (3.5 mg/kg total dose), for which a better efficacy has been demonstrated in six months compared with regimens shorter in 3 or 5 days of the same daily dose (RR 3.18 for the development of recurrences, 95% CI= 2.1-4.81 and RR= 10.05; 95% CI= 2.82-35.86, to schemes 3 and 5 respectively compared with the standard regimen) 10. The incidence of recurrences for the standard regimen after a supervised treatment varies from 0% to 32% with a follow-up between 90 and 365 days 11. This large variation can be attributed to the complexity of the assessment of therapeutic efficacy of this antimalarial where several factors are involved, such as the efficiency of the blood schizontocidal, the length of the follow-up, adjustment of the dose by body weight, drug absorption and metabolism, level of malaria transmission during the study (in case of endemic area), the relapse pattern of the *P. vivax *strain, the origin of the strain and the diagnostic method for evaluating recurrences 3,9. 

Although most endemic countries follow the WHO recommendation to use PQ in the first-line treatment of *P. vivax* malaria, some countries are currently using alternative regimens such as applying twice the standard daily dose (0.5 mg/kg/day) during 7 days (3.5 mg/kg total dose) or 14 days (7 mg/kg total dose) 2, the latter is recommended by the Center for Disease Control and Prevention (CDC) of the United States 12. Although changes in PQ treatment protocols have been implemented in order to improve adherence and reduce relapses, the efficacy and safety of these alternative regimens, compared to the standard regimen, is unknown. 

The primary objective of this systematic review was to compare the efficacy and safety of the PQ regimen of 0.5 mg/kg/day for 7 days with the standard regimen of 0.25 mg/kg/day for 14 days in patients infected with uncomplicated *P. vivax *malaria. The secondary objective was comparing the PQ regimen of 0.5 mg/kg/day for 14 days with the standard regimen.

## Materials and Methods

A protocol (unpublished) was designed following the recommendations of the Cochrane collaboration for systematic reviews and the PRISMA guide (Preferred reporting items for systematic reviews and meta-analyzes) for the reporting of the results was followed 13.

### Eligibility criteria 

Clinical trials with and without random allocation evaluating the regimen of 0.5 mg/kg/day for 7 or 14 days in participants diagnosed with uncomplicated *P. vivax* malaria and follow-up longer than 28 days. The main outcome was the cumulative incidence of recurrences after a 28-day follow-up since the first dose, defined as a *P. vivax* infection in patients who previously had an adequate clinical response to the treatment. As a secondary outcome, the treatment adverse events (rash, dizziness, headache, abdominal pain, nausea, jaundice, hemoglobin, hemoglobinuria, agranulocytosis, leocopenia, cyanosis, hypertension, cardiac arrhythmia) were assessed. 

### Searching strategy

Searches were performed on the following electronic data bases: MEDLINE, EMBASE, LILACS, SCIELO, the Central Register of Controlled Clinical Trials from the Cochrane Group and Clinical Trials. The search was done until August 20, 2015; unrestricted by language or publication date. The searching strategy in MEDLINE and EMBASE was: "(vivax malaria) OR (*Plasmodium vivax*) AND (recurrence OR recurren* OR relapse) AND primaquine". For searching other databases, the keywords "(vivax malaria) OR vivax AND primaquine" were used. The references of the selected articles from the electronic search and the systematic and narrative reviews published previously were reviewed. The European database Open Grey was revised, in search of unpublished studies using the term "primaquine". Finally, the available reports of recent events by the ASTMH Annual Meeting, of the Latin American Parasitology Convention and of the Convention of the Colombian Association of Parasitology and Tropical Medicine were revised; for searching in these reports, the term "primaquine" was used, or "primaquina" when the information was only available in Spanish.

###  Selection of studies

The eligibility of studies was assessed independently by two of the three authors of a standardized form. Disagreements were resolved by consensus between the three authors. In the screening phase, the titles and abstracts were revised, in a second phase, the full articles were reviewed, and finally the information extraction and quality assessment of the studies included was performed. 

###  Data extraction 

The information in each study was extracted by one author and subsequently confirmed by a second one; the information included was the year of publication, the place where the study was conducted (country and region), the number of arms, the intervention and the comparison group, the adjustment of the PQ dose according to body weight, the diagnosis test used for detect recurrence, if the follow-up of participants took place in an endemic area, the population type, if the parasitaemia was or was not an inclusion criterion, the performance of tests for glucose-6-phosphate dehydrogenase (G6PD), the simultaneous or non-simultaneous administration of the PQ and the blood schizonticide, the PQ daily dose, the treatment time, the total dose, the treatment supervision, the basal characteristics (age and gender), the number of participants, the length of follow-up, the absolute frequency of recurrences, the cumulative incidence of recurrence and the adverse events associated with the PQ treatment.

###  Risk of bias in individual studies

In order to assess the quality of the studies, the approach proposed by the GRADE (Grading of Recommendations Assessment, Development and Evaluation) group was followed 14. For this, the Cochrane tool was used, which includes a checklist with which the authors of this systematic review assessed, the random sequence generation, the allocation concealment, blinding of participants and personnel, the blinding of outcome assessment, the incomplete outcome data and the selective reporting of outcomes.

###  Summary measures and synthesis of results 

The cumulative incidence of recurrences (number of events during the follow-up period of all participants) was used as a summary measure for individual studies; besides, the relative risks ratio (RR) and their respective confidence intervals (95% CI) were calculated for studies that made the comparison with the standard regimen. For the primary objective, comparing the efficacy and safety of PQ 0.5 mg/kg/day for 7 days with the standard regimen, a meta-analysis of fixed effects was performed, with which the overall RR and its 95% CI was estimated. For the secondary objective, comparing the PQ regimen of 0.5 mg/kg/day for 14 days with the standard regimen, a meta-analysis could not be performed because only one study was found. The assessment of the heterogeneity of the results between studies was performed with the Chi Square test with a *p*< 0.10 value indicating heterogeneity, and the I^2^ statistic assuming significant heterogeneity if it was higher than 20%. 

## Results 

### Selection of studies

**Figure 1.**
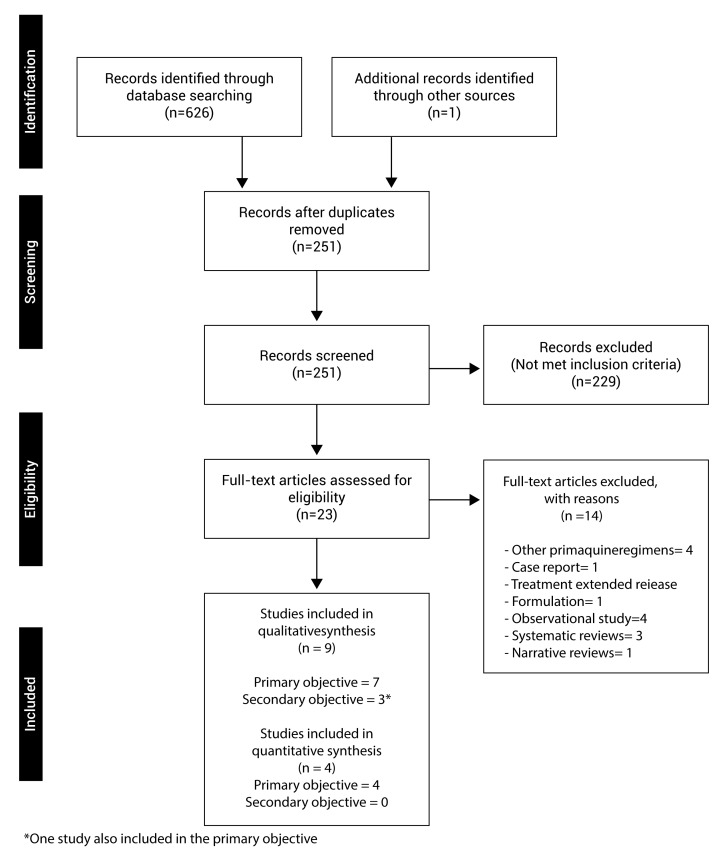
Flowchart of studies included. The results of the search process and the selection of studies to be included in this revision are shown.

A total of 626 articles were identified in the electronic databases, 213 in MEDLINE, 344 in EMBASE, 49 in LILACS, 28 in SCIELO, 103 in CENTRAL of Cochrane and 33 in Clinical Trials; and, additionally, an article was identified in other sources. 376 were duplicated and 251 articles were screened, out of which 229 were excluded. Twenty-three articles were fully read for review in the second phase, out of which 9 clinical trials met with the eligibility criteria; 7 studies for the qualitative systematic review of the primary objective 15-21, out of these, 4 studies made a comparison with the standard regimen and were included in the meta-analysis 15,17,19,20, the other 3 did not compare with the standard regimen 16,18,21. Three studies for the secondary objective were identified 19,22,23, one of which was also included in the primary objective; only one study made a comparison with the standard regimen 19, therefore, no studies for the quantitative synthesis of this objective were included (Fig. 1).

###  Characteristics of the studies 

Table 1 shows the main characteristics of the studies. Three studies were conducted in Brazil 15,16,18, two in Peru 17,20, one in Colombia 21, one in India 19, one in Pakistan 22, and one in Indonesia 23. Only one study was conducted in a non-endemic region where reinfection during the follow-up was not possible 23. The length of follow-up was variable with an average of 200 days (range= 60-365). 

**Table 1.** Characteristics of the studies included. The main characteristics of the studies regarding the place where they were conducted, the inclusion criteria for participants and the follow-up to assess recurrences is shown*.

Region	First author	Blood schizonticide + Intervention/comparison	Dose adjustment for body weight greater than 60 kg	Endemic region	Population	Inclusion criteria parasitemia diagnosis day (# parasites/µl)	G6PDH test before administering PQ	Simultaneous treatment blood schizonticide and PQ	Supervision treatment	Genoyping for clasiffication recurrences (relapse or reinfection)
America	Pinto, *et al* ^18^ Brazil	CQ 10 mg/kg single dose + PQ 0.5 mg/kg/day 7 days	Not applicable	Yes	Community, children between 0 and 15 years old	No	No	Not specified	Not specified	No
	Abdon, *et al* ^15^ Brazil	CQ 25 mg/kg 3 days + PQ 0.25 mg/kg/day 14 days	Not specified	Yes	Community, age higher than 12 years	No	Not specified	Not specified	Yes	No
		CQ 10 mg/kg single dose + PQ 0.5 mg/kg/day 7 days						Not specified	Yes	
	Solari-Soto, *et al* ^20^ Peru	CQ 25 mg/Kg + PQ 0.25 mg/kg/day 14 days	Not specified	Yes	Community, age higher than 12 years	No	Not specified	Yes	Yes	No
		CQ 25 mg/Kg + PQ 0.5 mg/kg/day 7 days							Yes	
	Da Silva, *et al* ^16 ^Brazil	AS 50 mg 0h and 12h + PQ 30 mg/d 7 days	No	Yes	Community, age higher than 14 years	No	Not specified	Not specified	Not specified	No
		AS 100 mg 0h and 12h + PQ 30 mg/d 7 days								
		AS 100 mg 0h and 50 mg 12h + PQ 30 mg/d 7 days								
		CQ 600mg single dose + PQ 30 mg/kg/d 7 days								
	Carmona-Fonseca, *et al* ^21^ Colombia	CQ 25mg/Kg in 3 days + PQ 210 mg in 7 days	Not specified	Yes	Community, children under18 years old	Yes; 1000 or more parasites/µl	Yes	Yes	Yes	No
	Durand, *et al* ^17^ Perú	CQ 25 mg / kg 3días + PQ 0,5 mg/kg/day 7 days	Not specified	Yes	Community, age higher than 1 year	Yes; between 250 - 100000 parasites/µl	Yes	Yes	Yes	Yes
		CQ 25 mg/kg 3 días + PQ 0,25 mg/kg/day 14 days	Not specified	Yes					Yes	
Southeast Asian	Sutanto, *et al* ^23^ Indonesia	Quinina (10 mg/kg 3 time by day, 7 days) + PQ 30 mg/day 14 days	Yes; maximum 45 mg/day, PQ in participant greater than 70 kg.	No	Soldiers	No	Yes	Yes	Not specified	Not applicable. All recurrences are relapse
	Dihidroartemisinina (120 mg) - piperaquina (960 mg)/day, 3 days + PQ after day 28; 30 mg/day, 14 days	No; PQ after finishing blood schizonticide	Not specified
	Rajgor, *et al* ^19^ India	CQ 25 mg/kg 3 days + PQ 15mg/day, 14 days	Not specified	Yes	Community, age higher than 18 years	No	Yes	No, PQ 24 hours after last CQ dosage	Yes	Yes
		CQ 25 mg/kg 3 days + PQ 30 mg/day, 7 days								
		CQ 25 mg/kg 3 days + PQ 30 mg/day, 14 days								
Eastern Mediterranean	Leslie, *et al* ^22^ 2008 Pakistan	CQ 25 mg/kg 3 days + PQ 0.5 mg/kg/day, 14 days	Not specified	Yes	Community, age higher than 3 years	No	Yes	Yes	Yes	No

G6PDH, Glucose 6-phosphate deshidrogenase, PQ, Primaquine, CQ, Chloroquine, AS, Artesunate

* All diagnosis test were made by microscopy

Clinical trials included a total of 1,996 participants, 1,486 received PQ 0.5 mg/kg/day for 7 days and 510 participants received PQ 0.5 mg/kg/day for 14 days. The participants were recruited from the civilian population in eight studies 15-22 and only one came from the military population 23. In five studies, they conducted the G6PD test before administering of the PQ 17,19,21-23, while others did not provide information about this test 15,16,18,20. In all studies microscopy was used as a diagnostic test for detecting recurrences of *P. vivax* malaria. Only two studies considered a parasitemia greater than 1,000 parasites /µL 21 or between 250 and 100,000 parasites /µL as an inclusion criteria 17. 

Seven clinical trials used PQ 0.5 mg/Kg/day for 7 days, in combination with a single dose chloroquine (CQ) of 10 mg/Kg 15,16,18, or CQ 25 mg/Kg for 3 days 17,19 -21 or artesunate in varied doses of 100, 150 and 200 mg during 7 days 16. Three studies evaluated PQ 0.5 mg/Kg/day for 14 days, in combination with quinine 10 mg/Kg 3 times a day for 7 days 23 or dihydroartemisinin (120 mg) plus piperaquine (960 mg) daily during 3 days (23) or CQ 25 mg/Kg for 3 days 19,22. In all studies, the maximum PQ dose was 30 mg per day, except in the study by Sutanto *et al.*, where a maximum of 45 mg per day were administered to participants whose body weight was over 70 Kg 23.

The regimens used to compare the PQ 0.5 mg/Kg/day for 7 or 14 days were heterogeneous; three studies used an arm without PQ for comparison 19,22,23, out of these, two used CQ 25 mg/Kg as blood schizonticide 19,22 and one used artesunate 23; four studies used the standard regimen of PQ for comparison and CQ was also used in them 15, 17, 19, 20, PQ 0.75 mg/Kg/week during 8 weeks was used in another one 22, while lower doses of PQ than standard regimen were used in the remaining studies 16,18,21. 

The percentage of cumulative incidence of *P. vivax* malaria recurrences was the primary outcome in all clinical trials included. Eight studies reported the assessment of adverse events to treatment 15-17,19-23 and only one study was unclear whether this outcome was measured or not 18. 

###  Bias risk of the studies 

**Figure 2.**
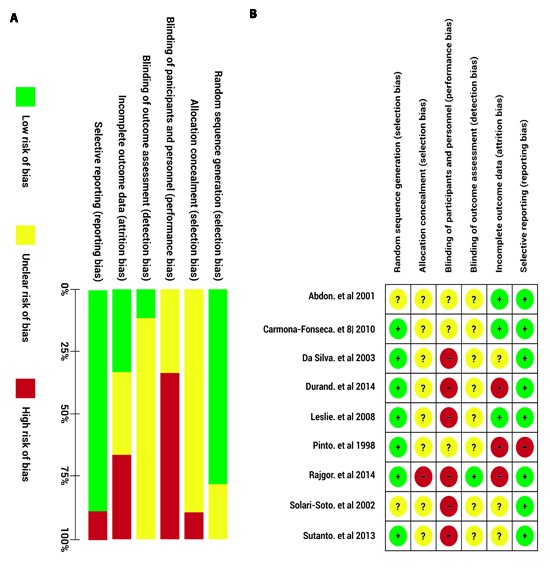
Summary of the authors' opinion about the bias risk in the studies included. The bias risk is grouped in seven domains assessing the potential bias sources, for each study, these domains were rated as low bias risk (green), high bias risk (red) or unknown bias risk (yellow) a) Bias risk of the individual studies for each item assessed. b) Global bias risk, presented as percentage of all the studies.

Figure 2a shows the judgment of the authors regarding the items assessed about methodological quality for each of the included studies. Figure 2b summarizes the overall bias risk for all clinical trials, the high risk, low risk or non-specified risk percentage in the study is represented for each item. None of the studies included was blinded to the participants nor considered allocation concealment; therefore, the bias risk for these items is high. A high bias risk in blinding those who evaluated the outcome and in management of losses during the follow-up was also found. The items assessed with the best methodological quality were the sequence generation for treatment assignment and the selective reporting bias of outcomes; the majority of studies generated the sequence through of a random number chart and considered the assessment of other outcomes such as adverse effects to treatment. 

###  Results of individual studies 

Table 2 shows the results for each study. A cumulative recurrence incidence mean of 8.41% (range = 0%-22.80%) was found for PQ 0.5 mg/Kg/day for 7 days 15-21 in a follow-up period from 90 to 210 days; and 8.38% (range= 1.90%-19.40%) was found for PQ 0.5 mg/Kg/day for 14 days from 180 to 365 days. For clinical trials comparing with the standard regimen, the recurrence mean was 7.58% (range= 0-10.20%) for PQ for 7 days, 6.60% for PQ for 14 days and 8.32% (range= 5.00%-13.50%) for the standard regimen.

**Table 2.** Results of the studies included. The main results of the studies regarding the population characteristics, the main outcome (recurrence) and the secondary outcomes (adverse effects to treatment) are shown.

Region	Reference	Daily PQ dose (mg/kg/day)	Duration PQ treatment (days)	Total PQ dose (mg/Kg)	Age (yrs)	Sex, percentage of men	Sample size	Length of follow-up (days)	Absolute recurrence frequency	% Cumulative recurrence incidence	Adverse effects
America	Pinto, *et al* *^18^*	0.5	7	3.5	rank 0 -15	Not specified	46	180	3	7,7	Not specified
	Abdon, *et al* *^15^*	0.25	14	3.5	mean 27 (IQR 12 - 67)	60	40	180	2	5	27.5% from PQ standar regimen and 15.4% from PQ 0.5 mg/Kg/día/7 días. Diarrhea (5.8%). Sickness (5%). Pruritus (5%). Vomiting (1.7%). Epigastric pain (1.7%). Dizziness (0.8%). buzz (0.8%)
		0.5	7	3.5		65	40	180	0	0	
	Solari-Soto, et al^20^	0.25	14	3.5	mean 28.9 (SD 17.2)	66.7	30	60	2	7	Not reported
		0.5	7	3.5	mean 24.0 (SD 17.0)	46.7	30	60	3	10	
	Da Silva, *et al* *^16^*	0.5	7	3.5	mean 31.8	93.3	30	180	0	0	Adverse effects were reported but not frequently specified in each arm, no differences between groups was reported. Pruritus 5.% and coluria 1.3%
		0.5	7	3.5	mean 33	66.7	30	180	1	3.3	
		0.5	7	3.5	mean 36.9	83.3	30	180	5	16.7	
		0.5	7	3.5	mean 36.6	70	30	180	1	3.3	
	Carmona-Fonseca, *et al* ^21^	0.5	7	3.5	rank 10 - 17	Not specified	41	120	8	22.8	Sweating 11% . Conjunctival pallor8.7%. Diarrhea8.1%. Pruritus7.6%. Sickness 7.6%. Dizziness7.0%. Vomit 6.4%. Abdominal pain5.8%. Anorexia3.5%.
	Durand, *et al* ^17^	0.5	7	3.5	mean 17.6 (rank 2-72)	52.8	180	210	16	10.2	Not reported
		0.25	14	3.5	mean 21.0 (rank 1-70)	53.6	180	210	22	13.5	
Southeast Asian	Sutanto, *et al* ^23^	0.5	14	7	mean 27.1 (IQR 22.1 - 31.5)	100	39	365	7	19.4	Not reported
	0.5	14	7	mean 27.8 (IQR 23.6 - 42.1)	100	36	365	2	5.6
	Rajgor, *et al* ^19^	0.25	14	3.5	mean 31 (SD 12)	94.9	398	180	26	8.1	5%
		0.5	7	3.5	mean 32 (SD 13)	95.5	381	180	30	10.1	10%
		0.5	14	7	mean 32 (SD 11)	96.0	380	180	21	6.6	13%
Eastern Mediterranean	Leslie, *et al* ^22^	0.5	14	7	meann 10 (IQR 4 - 45)	44.0	55	330	1	1.9	Not reported

PQ, Primaquine, IQR, Interquartile range, SD, Standard deviation

### Synthesis of results 

**Figure 3.**
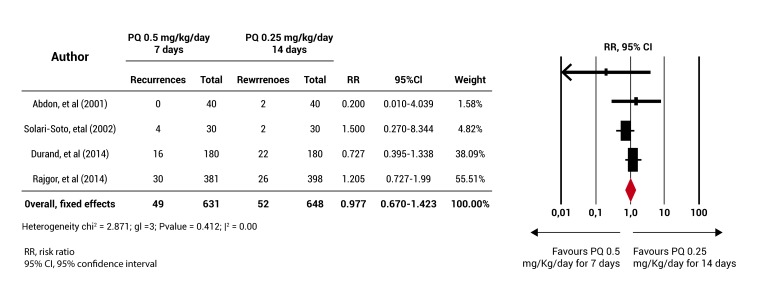
Meta-analysis results. The diagram represents the number of participants and the total number of events (recurrences) for the group receiving the primaquine 0.5 mg/Kg/day for 7 days and for the group receiving the primaquine 0.25 mg/Kg/day for 14 days (standard regimen). The ratio relative risks (RR) are shown with their respective confidence intervals (IC 95%) and the meta-analysis performed.

For the primary objective, a meta-analysis was performed, which showed that the rate of PQ 0.5 mg/kg/day for 7 days does not have an efficiency lower than that of the standard regimen (RR= 0.977 (95% CI= 0.670-1.423) Fig. 3. Although no statistical heterogeneity among the studies included in this analysis was found, the four clinical trials have a different length of follow-up 15,17,19,20 and these also showed a high bias risk in some of the items assessed for methodological quality. It was not possible to conduct sensitivity analyzes (according to the random allocation of the intervention, length of follow-up and bias risk) and the subgroup analysis (according to the blood schizontocide used, the age group and population type) that had been planned due to the small number of included studies.

For the secondary objective, only one study compared PQ 0.5 mg/Kg/day for 14 days with the standard regimen 19, the RR of recurrence after 180-day follow-up was 0.846 (95% CI= 0.484-1.477). It was not possible to perform a meta-analysis.

## Discussion

A priority for the control and elimination of malaria in the world is having an effective treatment to prevent *P. vivax* relapses 24. Some previous systematic reviews have compared the therapeutic efficacy of the standard PQ regimen (0.25 mg/Kg/day for 14 days) mostly used to prevent recurrences of *P. vivax* malaria with other regimens. Including studies until 2012, they have shown that the total doses lower than the standard regimen have lower efficacy 10,11,25; however, none of the previous systematic revisions have focused on evaluating the effectiveness of both PQ regimens currently used in the world as an alternative to improve efficiency or adherence to the standard PQ regimen (0.5 mg/Kg/day for 7 and 0.5 mg/Kg/day for 14 days); therefore, this systematic revision has addressed this comparison.

We only included trials with a follow-up longer than 28 days, we identified four clinical trials that compared the standard PQ regimen with the PQ regimen of 0.5 mg/Kg/day for 7 days, and only one study comparing with the PQ regimen of 0.5 mg/Kg/day for 14 days. Additionally, we identified 5 clinical trials where one of these two alternative regimens was assessed, but without the comparison with the standard regimen. This systematic review could omit other studies, because here we only consider some of the search sources different from electronic databases (European Open Grey, reference search in the clinical trials registry and search some reports of academic events,) but the search for abstracts in other malaria-related conferences and contact with investigators to try to identify other unpublished or ongoing studies was not included.

Considering that recurrences can occur in less than 28 days after the primary episode 3-5, it is possible that some studies were omitted; however, we limit the systematic revision of studies with a follow-up longer than 28 days since most treatment regimens used for *P. vivax* malaria use at least one long half-life blood schizontocidal such as chloroquine; and generally the therapeutic response to them is assessed during the first post-treatment month 7,8,26; therefore we did not include these studies in order to avoid an overestimation of the incidence of recurrences that could be caused by a recrudescence of blood parasites (blood schizonticide therapeutic failure) and not due to the lack PQ efficacy. 

We observed a mean of 8.38% of recurrence incidence for the regime which doubles the total dose of PQ, i.e. 0.5/mg/Kg/day for 14 days; this value was similar to the mean for the standard regimen as reported in a previous systematic revision (9.9%) 11; and it is even apparently equal to the mean found for the PQ regimen of 0.5 mg/Kg/day for 7 days (8.41%). We attribute these results to the heterogeneity between studies, although the Chi^2^ and I^2^ statistical test did not show such heterogeneity possibly due to the lack of power, important differences were found regarding the length of follow-up, the time when the study was conducted, the blood schizontocides used, in addition to the deficiencies in the conduction of studies that cast doubt on the validity of the comparisons. 

Another source of heterogeneity in the results could be the origin of the participants, since there are different patterns of relapse by geographic region 3-5; we include clinical trials from three different geographical regions (The Americas, Southeast Asia and Eastern Mediterranean) for which it has been reported that the most common relapse pattern is that one of short intervals; therefore, we believe that the recurrence incidence found for the PQ in this systematic revision may be higher than that one that might be found in other regions such as Europe or Africa where the pattern of late relapse is more common 3. Although there is insufficient knowledge about the distribution of *P. vivax* strains according to the relapse pattern, there is some evidence of the simultaneous presence of several patterns in the same region 3,27 which may explain the variability found for studies in the region of the Americas included in this revision.

The evidence found in this systematic review is considered to have low quality due to the high bias risk identified in the clinical trials included or due to the lack of clarity for the items considered in the methodological quality assessment 14, this is the main aspect supporting the lack of evidence to compare the PQ regimens currently used and choose the best regimen to prevent *P. vivax* relapses. Most studies had methodological limitations in the allocation concealment, in the blinding of participants and staff evaluating the outcome, and in the management of incomplete data; these limitations could bias results favoring efficacy (lower recurrence incidence) of the PQ regimen of 0.5 mg/Kg/day for 7 or 14 days in relation to the standard regimen.

Eight out of the nine clinical trials included in this review assessed adverse effects to treatment, these considered mainly abdominal pain, nausea, vomiting, itching, dizziness, epigastric pain, hemolysis and methamoglobinemia, which are the major side effects for the PQ 7. Some studies possibly did not provide further details of adverse events to treatment because of its low frequency in patients with normal activity of the G6PD enzyme, which was one of the inclusion criteria to administer the PQ in most of the studies included.

During the conduction of this systematic revision we identified 4 prospective observational studies evaluating PQ regimens of our concern 28-31. One study evaluated the efficacy of the PQ regimen 0.5 mg/Kg/day for 14 days using atovaquone-proguanil as blood schizonticide 29; in this study a recurrence incidence by 5% in 84 days was reported, similar to what we found in clinical trials with this PQ regimen. Three observational studies for the PQ regimen of 0.5 mg/Kg/day for 7 days plus a CQ regimen of 25 mg/Kg during 3 days 28,30,31, which showed conflicting clinical trials included in this review were found; the recurrence incidence for these was between 26% and 76% with a follow-up between 180 and 720 days. This large difference, besides from being explained by methodological limitations and lack of uniformity in follow-up time, they are explained by differences in the method for detecting recurrences; 2 observational studies included a molecular diagnosis (PCR) to assess the outcome 28,31), because this technique has a detection limit lower than that of microscopy 32,33, sub-microscopic and asymptomatic recurrences were found in these studies, showing reduced efficacy of PQ. 

Only two studies looked at the genetic characterization of recurrences to be classified as relapses or reinfections 17,19 in both studies, less than 50% of recurrences were classified as relapses. Although the methods selected for genotyping have limitations, this finding is highlighted to emphasize the importance of including the genetic characterization of *P. vivax* in the studies intending to assess recurrences, since it could contribute to a better approach to the therapeutic efficacy of PQ. 

## Conclusion

The PQ regimen of 0.5 mg/Kg/day for 7 or 14 days is currently used in some countries instead of the standard treatment to prevent *P. vivax* relapses, though here it was found that apparently the 7-day regimen is not inferior than the standard one, this systematic revision suggests that given the small number of clinical trials reported and methodological limitations thereof, there is insufficient evidence to determine which PQ regimen used as first-line treatment for *P. vivax *in the world has the best efficacy and safety in preventing relapses. It is necessary to conduct clinical trials with a high methodological quality to compare these regimens and thus have therapeutic alternatives to prevent relapses, which may guide changes in treatment protocols for *P. vivax* malaria.
